# Identification of immune-related endoplasmic reticulum stress genes in sepsis using bioinformatics and machine learning

**DOI:** 10.3389/fimmu.2022.995974

**Published:** 2022-09-20

**Authors:** Ting Gong, Yongbin Liu, Zhiyuan Tian, Min Zhang, Hejun Gao, Zhiyong Peng, Shuang Yin, Chi Wai Cheung, Youtan Liu

**Affiliations:** ^1^ Department of Anesthesiology, Shenzhen Hospital, Southern Medical University, Shenzhen, China; ^2^ The Third School of Clinical Medicine, Southern Medical University, Guangzhou, China; ^3^ Department of Radiology, Huazhong University of Science and Technology Union Shenzhen Hospital, Shenzhen, China; ^4^ Department of Anesthesiology, The University of Hong Kong, Hong Kong, Hong Kong SAR, China

**Keywords:** sepsis, immunity, endoplasmic reticulum stress, machine learning, SCAMP5

## Abstract

**Background:**

Sepsis-induced apoptosis of immune cells leads to widespread depletion of key immune effector cells. Endoplasmic reticulum (ER) stress has been implicated in the apoptotic pathway, although little is known regarding its role in sepsis-related immune cell apoptosis. The aim of this study was to develop an ER stress-related prognostic and diagnostic signature for sepsis through bioinformatics and machine learning algorithms on the basis of the differentially expressed genes (DEGs) between healthy controls and sepsis patients.

**Methods:**

The transcriptomic datasets that include gene expression profiles of sepsis patients and healthy controls were downloaded from the GEO database. The immune-related endoplasmic reticulum stress hub genes associated with sepsis patients were identified using the new comprehensive machine learning algorithm and bioinformatics analysis which includes functional enrichment analyses, consensus clustering, weighted gene coexpression network analysis (WGCNA), and protein-protein interaction (PPI) network construction. Next, the diagnostic model was established by logistic regression and the molecular subtypes of sepsis were obtained based on the significant DEGs. Finally, the potential diagnostic markers of sepsis were screened among the significant DEGs, and validated in multiple datasets.

**Results:**

Significant differences in the type and abundance of infiltrating immune cell populations were observed between the healthy control and sepsis patients. The immune-related ER stress genes achieved strong stability and high accuracy in predicting sepsis patients. 10 genes were screened as potential diagnostic markers for sepsis among the significant DEGs, and were further validated in multiple datasets. In addition, higher expression levels of SCAMP5 mRNA and protein were observed in PBMCs isolated from sepsis patients than healthy donors (n = 5).

**Conclusions:**

We established a stable and accurate signature to evaluate the diagnosis of sepsis based on the machine learning algorithms and bioinformatics. SCAMP5 was preliminarily identified as a diagnostic marker of sepsis that may affect its progression by regulating ER stress.

## Introduction

Sepsis is associated with high morbidity and mortality rates which caused by a disproportionate inflammatory response of the host to infection (1). An estimated 48.9 million people worldwide were diagnosed with sepsis in 2017, resulting in over 11 million deaths that accounted for 20% of the global mortality rate (2). Despite advances in resuscitation strategies, ventilator management, antibiotic therapy and glucose maintenance, there is no particularly effective treatment for sepsis other than standard care and supportive treatment, and severe sepsis remains a leading cause of death (3, 4). Studies in human subjects and animal models have shown that sepsis is associated with the overactivation of innate immune effector cells, resulting in uncontrolled inflammation that leads to extensive tissue damage and organ failure in case of severe septicemia (5–7). In order to reduce sepsis-related mortality, it is very necessary to explore the biological mechanisms and potential biomarkers associated with sepsis.

Endoplasmic reticulum (ER) is the place of protein folding and post-translational modifications, and is also a critical organelle of the secretory pathway (8). Cellular stress and inflammation can lead to the accumulation of unfolded or misfolded proteins, a phenomenon also known as ER stress (9). ER arising from inflammation and the loss of dynamic balance in endoplasmic reticulum function under stress has been closely related to the progression of sepsis (10). However, the possible relationship between ER and sepsis, especially the possible role of ER stress on immune cell apoptosis during sepsis, remains unclear. To this end, we explored the role of immune cell apoptosis and ER stress on the development of sepsis, as well as their correlation to patient prognosis. Our objective was to identify the molecular subtypes of sepsis to expand the repertoire of potential diagnostic biomarkers.

The gene expression profiles of sepsis and normal blood samples were retrieved from the GEO database using R software (11), and the differentially expressed genes (DEGs) between the two groups were screened. Immune cell infiltration in the sepsis and control groups was analyzed using the CIBERSORT algorithm, and the sepsis dataset was clustered on the basis of immune checkpoint genes in order to identify key genes associated with the immune responses during sepsis. The DEGs related to sepsis and ER stress were functionally annotated by GO and KEGG pathway enrichment analyses, and weighted gene correlation network analysis (WGCNA) (12) was performed to identify co-expressed gene modules. Next, the protein-protein interaction (PPI) network of the genes intersecting the WGCNA and ER stress-related gene sets was constructed using the STRING database (13), and the clinical relevance of the hub genes was analyzed in multiple datasets. In addition, the correlation between the hub genes and immune cell infiltration levels was also examined. Finally, the potential diagnostic markers of sepsis were screened, which offers new insights for sepsis diagnosis and treatment.

## Materials and methods

### Data availability

All the raw data is available.

Raw data link: https://www.jianguoyun.com/p/DU2vz6oQzM3iChj1us0EIAA.

(Access Password: k6zrvo).

### Identification of sepsis-related DEGs

The sepsis-related transcriptomic datasets GSE9960 and GSE57065 (14, 15) were downloaded from the GEO database using the GEO query package in R (version 4.0.3, http://r-project.org/) (16). The details of the datasets are listed in **Table 1**. The datasets were merged using the sva package in R, and the difference between batches was eliminated according to the data source. The samples in the merged dataset were divided into the normal (n = 41) and sepsis (n = 136) groups using ComBat in the sva package, and all samples were included in the study. After normalizing the expression data, the DEGs between the normal and sepsis samples were screened by the limma package in R (17), with logFC > 1 or < -1 and adjP value < 0.05 as the thresholds.

**Table 1:** Data information.

Data	Normal	Sepsis
GSE9960	16	54
GSE57065	25	82
GSE123729	11	15
GSE54514	18	35
GSE26378	21	82

### Analysis of immune infiltrating cells in sepsis

Based on the principle linear support vector regression, we used CIBERSORT algorithm to analyze the gene expression matrix of immune cell subtypes. LM22 and CIBERSORT matrices can predict the proportion of 22 infiltrating immune cell subtypes in individual samples of a dataset (18). The infiltrating immune cell populations in the sepsis and normal samples were estimated on the basis of RNA-Seq data, and the abundance of the 22 subtypes of immune cells in the datasets was evaluated by the CIBERSORT algorithm. The differentially enriched immune cells between septic and normal samples were also identified, and their correlation with key sepsis-related genes was analyzed.

### Identification of immune subtypes

Consensus Clustering is used to determine the number of possible clusters in gene expression datasets, and is routinely applied in cancer genomics research to identify molecular subtypes. The “ConsensusClusterPlus” package in R (19) was used to cluster the sepsis datasets on the basis of immune checkpoint genes (20) in order to distinguish immune subtypes and identify the key genes related to sepsis-related immunity. The number of clusters was set between 2 and 10, and the process was repeated 100 times to extract 80% of the total samples using clusterAlg = “pam”, distance = “Euclidean”. The pheatmap package in R was used to draw the clustering heat map consisting of the top 20 down-regulated and up-regulated genes.

### Functional annotation of DEGs

Gene ontology (GO) is used for large-scale functional annotation of genes based on the enriched molecular functions (MF), biological processes (BP) and cellular components (CC). Subsequently, KEGG is a database of biological pathways, drugs, genomes and diseases. The clusterProfiler package in R (21) was used for KEGG pathway enrichment analyses and GO functional annotation of the intersecting sepsis-related DEGs and ER stress-related genes. P-value < 0.05 was used as the threshold for significant enrichment. Gene set enrichment analysis (GSEA) is used to evaluate the correlation of genes in a pre-defined gene set with a specific phenotype (22). The “c5.go.v7.4.symbols” with “c2.kegg.v7.4.symbols”gene sets in the MSigDB database (23) were subjected to GSEA using the clusterProfiler package (21). P-value < 0.05 was considered statistically significant (23).

### Weighted Gene Correlation Network Analysis (WGCNA)

WGCNA is used to identify co-expressed gene modules, explore the relationship between gene network and phenotype, and study the core genes in the network. WGCNA was performed on the DEGs between sepsis and control datasets using the WGCNA package in R (12). The correlation coefficient between two genes was first calculated, then its weighted value was used to make the connection between the genes in a scale-free network. The hierarchical clustering tree was then constructed according to the correlation coefficients, wherein different gene modules were represented by the branches and color-coded. The “minModuleSize” was set to 50, and the module significance and correlation of mRNA expression levels with different modules were calculated. Finally, the most significant module related to the disease was identified, and the characteristic genes were extracted for subsequent analysis.

### Construction of protein-protein interaction (PPI) networks

The STRING database (13) contains 2031 species, which includes 9.6 million proteins and 1380 million protein and protein interactions (PPIs) obtained from experimental data, text mining results from PubMed, other databases, and bioinformatics predictions. The PPI network of the genes intersecting the WGCNA and ER stress-related gene sets was visualized using Cytoscape software which constructed from the STRING database. Finally, the hub genes related to ER stress in sepsis were screened from this PPI network.

### Construction of a diagnostic model

The minor absolute contraction and selection operator (LASSO) logistic regression method is used to screen for the most powerful prognostic predictors since it forces the absolute value of the regression coefficient to be less than the constant value, which can effectively avoid model overfitting and filter out the most important events. The sepsis-related genes were preliminarily screened by the LASSO method using glmnet package in R (24), and the diagnostic model was established by logistic regression. The odds ratio (OR) and P-value of each variable were calculated in the model, then the risk score of each sample was obtained. Diagnostic marker genes with a P-value < 0.05 and OR value that is more excellent than or less than one were selected.

### Classification of sepsis subtypes

We used the “limma” package in R to screen the differentially expressed genes in the combined datasets between normal and sepsis samples. The filtering conditions were | logFC | > 2 and adj.P Value<0.05. The ConsensusClusterPlus package in R (19) was used to cluster the sepsis datasets based on the significant DEGs between sepsis and control samples to obtain molecular subtypes of sepsis.

### Extraction of peripheral blood mononuclear cells (PBMCs)

The collection of blood samples from human subjects was approved by the Medical Ethics Committee of Shenzhen Hospital of Southern Medical University (ID: NYSZYYEC20200039). The clinical data is available at the China Clinical trial Registration Center (No. ChiCTR2100043761). Healthy volunteers were recruited from hospital staff and through advertisements. All sepsis patients had been admitted to the ICU of the Shenzhen Hospital of Southern Medical University. The Third International Consensus Definitions for Sepsis and Septic Shock (Sepsis-3) were used to diagnose sepsis (25). Blood samples were collected by venipuncture, and the PBMCs were separated by Ficoll-Paque density gradient centrifugation as per the manufacturer’s instructions.

### Real-time quantitative PCR

RNA was extracted from cells and tissues using TRIzol (Gene Copoeia, MD, USA), and 1 µg total RNA from each sample was reverse transcribed to cDNA using specific primers and SYBR Green reaction mix (Takara Biotech). Real-time qPCR was performed on the Bio-Rad Real-Time PCR cycler. Relative gene expression levels were calculated by the 2^-ΔΔct^ method. The primer sequences were as follows: SCAMP5 forward: GCCCCATCAAGGTTCAGGAC, reverse: TACGTGTAATTGGGGGTGGC; GAPDH forward: TGGTATCGTGGAAGGACTC, reverse: AGTAGAGGCAGGGATGATG.

### Western blotting

After proteins quantified by a BCA protein assay kit (Thermo), equal amounts of proteins (20μg) per sample were separated by 10% SDS-PAGE and transferred to a PVDF membrane (Millipore, Billerica, MA, USA). After blocking with 5% skimmed milk at room temperature for 2 h, the membranes were incubated overnight with the anti-SCAMP5 (Abcam, ab3432, 1:500) and anti-GAPDH (Abcam, ab22555, 1:1000) primary antibodies, and thereafter with the horseradish peroxidase (HRP)-conjugated secondary antibody. The images were captured using the ChemiDoc imaging system (Bio-Rad).

### Statistical analysis

All statistical analyses were conducted using R (https://www.r-project.org/, 4.0.2 version). Normally distributed continuous variables between two groups were compared by the independent Student t-test, and variables with non-normal distribution were analyzed by the Mann-Whitney U test (Wilcoxon rank-sum test). The receiver operating characteristic curve (ROC) was plotted to predict binary categorical variables using the pROC package. All statistical tests were two-sided. P < 0.05 was regarded as statistically significant.

## Results

### Screening for DEGs between sepsis and control samples

**Figure 1:**
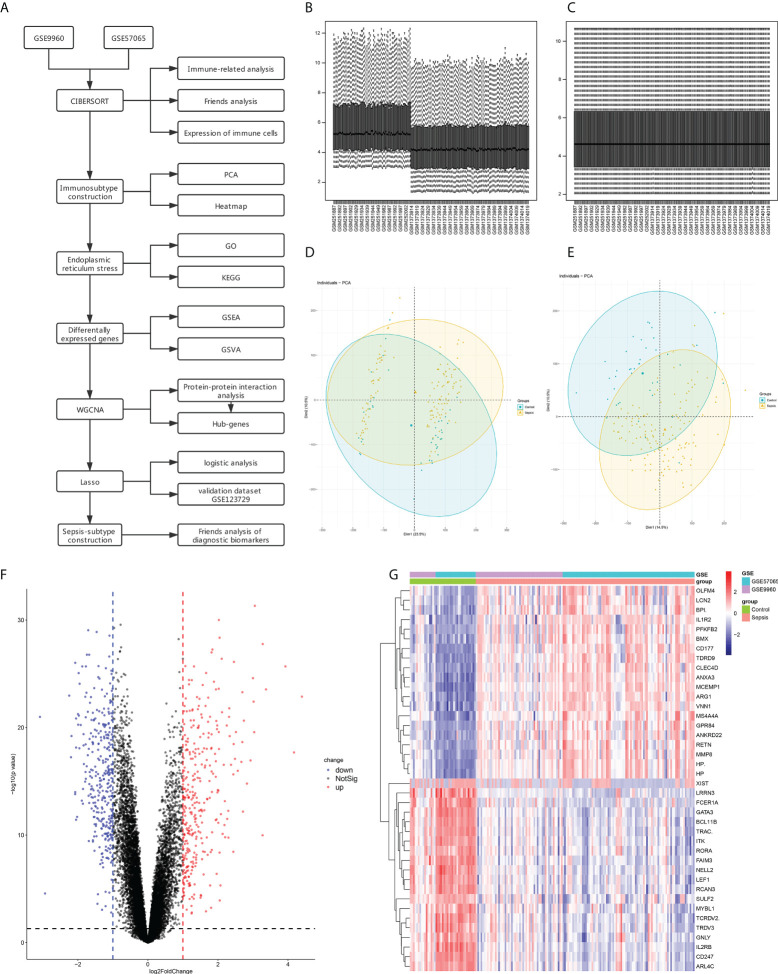
Data Preprocessing and identification of differentially expressed genes (DEGs). **(A)** Flow chart for gene set analyses. **(B)** Box line diagram of the merged dataset before correction. **(C)** Box line diagram of the combined dataset after correction. **(D)** PCA for sepsis and healthy control samples before batch correction with ComBat. **(E)** PCA for sepsis and healthy control samples after batch correction with ComBat. **(F)** Volcano plot showing DEGs between sepsis and control samples. **(G)** Heatmap showing the top 20 up- and down-regulated genes.

Data set analysis and flow chart of this study (**Figure 1A**). The GSE9960 and GSE57065 datasets were merged and batch effects were removed. To ascertain any significant differences in the expression profiles of the two datasets, we analyzed data distribution before and after removing the batch effect through box plots. As shown in **Figures 1B**, **D** there were apparent inter- and intra-group differences before removing the batch effect, which were eliminated once the batch effect of the dataset source was removed and corrected (**Figures 1C, E**). The DEGs between the sepsis and control groups were then screened using limma in R, which revealed 577 DEGs, including 325 up-regulated and 330 down-regulated genes (**Figures 1F, G**).

### Analysis of immune cell infiltration

**Figure 2:**
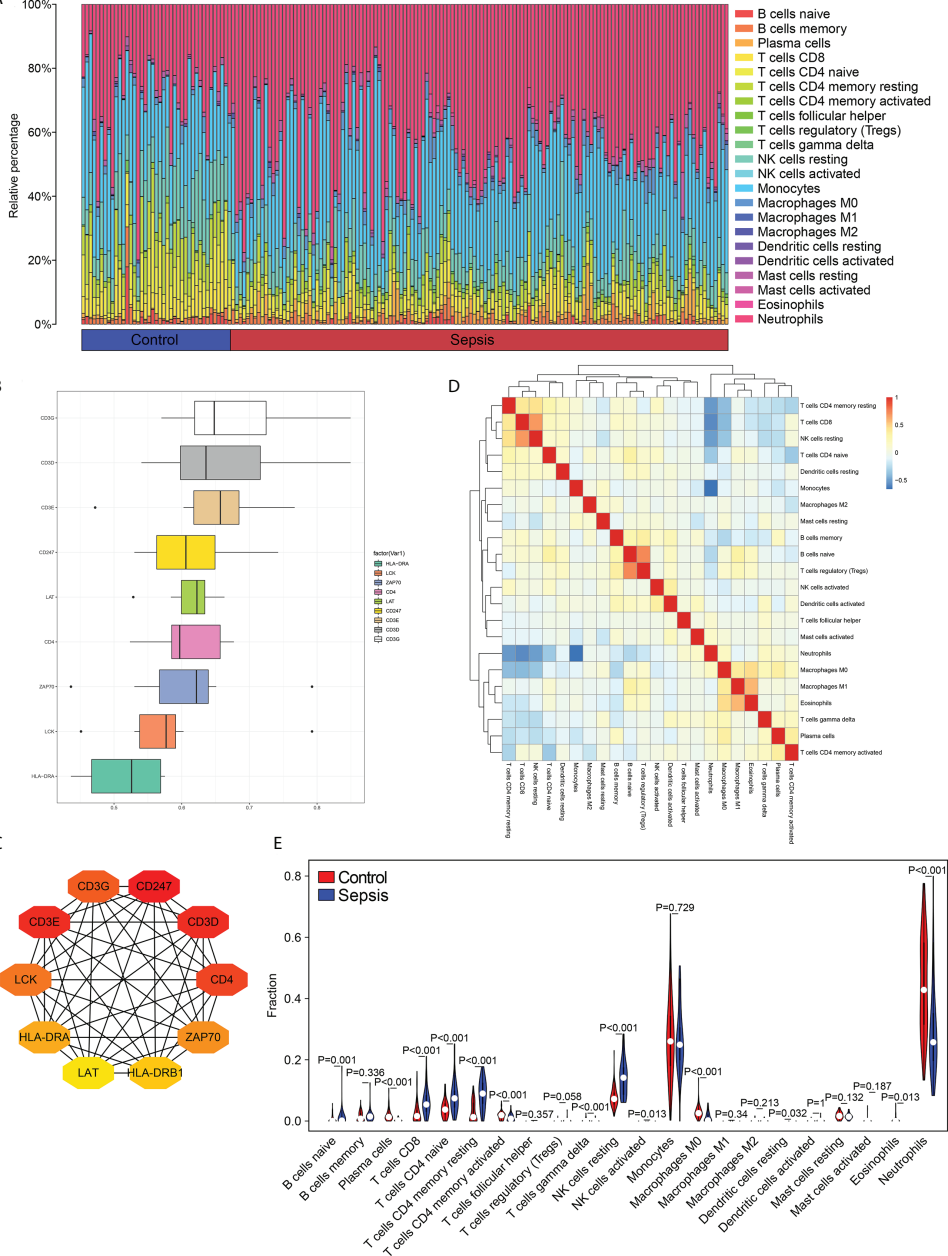
Distribution of immune cell subtypes in the merged dataset. **(A)** Bar plot showing percentage infiltration of 22 immune cells in each sample. **(B)** The top 10 hub genes according to Friends analysis. **(C)** The PPI network shows the interactions of the top10 genes. **(D)** Correlation heatmap of 22 immune cell types. **(E)** Violin plot showing differential infiltration of the 22 immune cell populations.

The proportion of different infiltrating immune cell types between the sepsis and control groups was evaluated using the CIBERSORT algorithm. After removing populations with a sum of immune abundance value 0, the Wilcox test algorithm was applied to 15 immune cell populations, including naïve B cells, plasma cells, memory B cells, CD8^+^ T cells, regulatory T cells (Tregs), CD4^+^ memory resting T cells, follicular helper T cells, resting NK cells, activated NK cells, M0 macrophages, M2 macrophages, monocytes,activated DCs, resting dendritic cells (DCs), resting mast cells and activated mast cells (**Figure 2A**).

To assess the functional correlation between key genes and immune cells in sepsis, we analyzed the PPI network of the 577 DEGs, and obtained the top 10 hub genes using the MCC algorithm, and carried out with Friends analysis (**Figure 2B**). The protein-protein interaction (PPI) networks for the top10 hub gene (**Figure 2C**). The correlation between immune cells in the datasets, and the abundance of different populations in the sepsis and control samples were analyzed. As shown in **Figures 2D, E**, the B cells, T cells, NK cells and DCs were more abundant in the sepsis samples compared to the controls, whereas the infiltration of neutrophils was significantly lower in the sepsis samples relative to that in the control samples. These findings indicate that the samples from normal and sepsis patients demonstrated a variety of different immune contexts.

### Identification of immune subtypes

**Figure 3:**
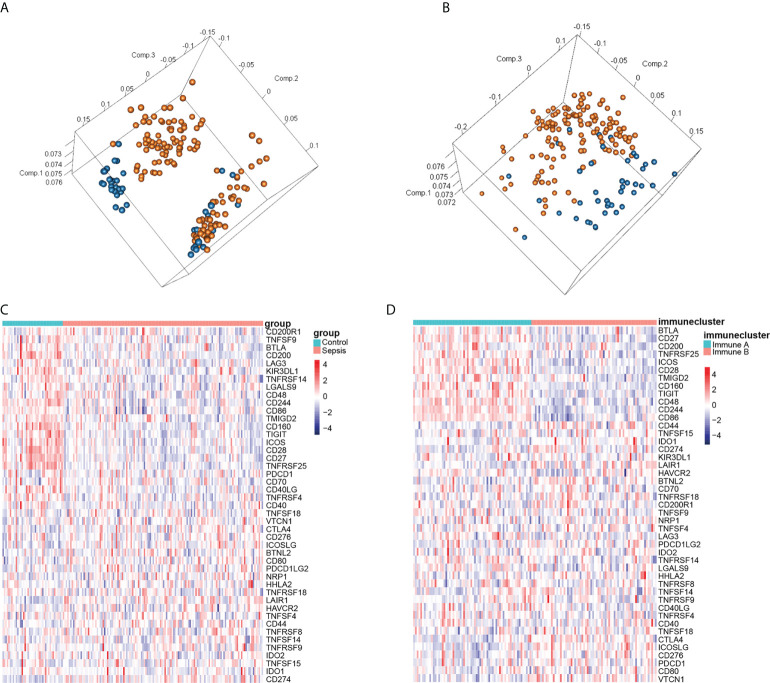
Identification of immune subtypes in sepsis. **(A)** PCA according to the subgroups of sepsis and healthy control samples. **(B)** PCA according to immunophenotyping. **(C)** Heatmap of immune infiltration-related genes in the normal and septic groups. **(D)** Heatmap of immune infiltration-related genes according to immunophenotyping. Red and blue squares indicate activation and suppression, respectively.

Principal component analysis (PCA) of the combined dataset showed that although the control and sepsis groups were distinct, there was still some overlap among the samples (**Figure 3A**). Since the immune checkpoint-related genes were differentially expressed between the sepsis and control groups (**Figure 3C**), we clustered the 136 sepsis samples on the basis of these immune checkpoints into the immune_ A (n = 66) and immune_ B (n = 70) clusters using the ConsensusClusterPlus package in R. PCA analysis was performed again (**Figure 3B**), and the results showed that although a small number of samples overlapped, most pieces were significantly separated. Next, we performed the differentially expressed genes just obtained to draw the heat map (**Figure 3D**), and the results show that the expression difference trend of these genes is more prominent. These findings indicated that sepsis samples were clustered into immune subsets based on immune checkpoint related genes were differentially expressed.

### Functional annotation of ER stress-related genes in sepsis

**Figure 4:**
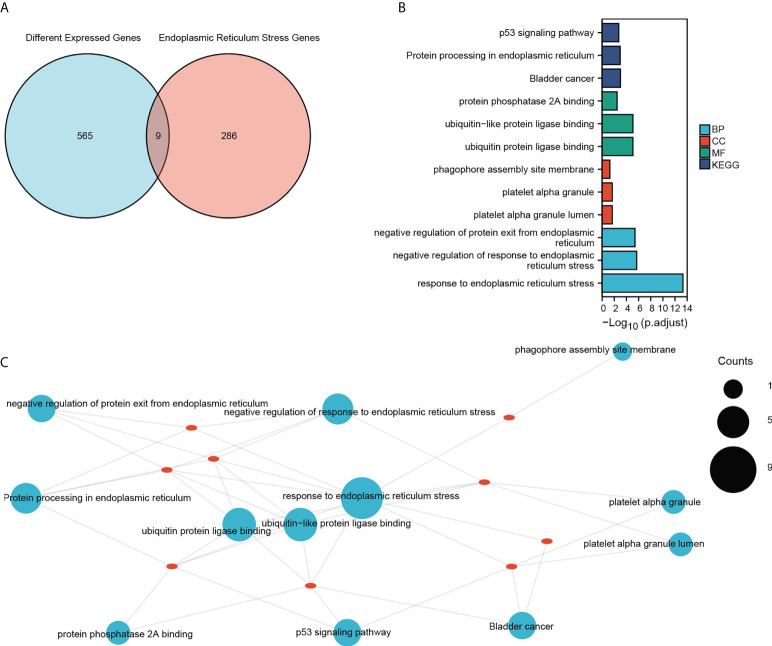
GO and KEGG enrichment analysis. **(A)** Venn diagram showing the intersection of DEGs and ER stress-related genes in the combined dataset. **(B)** GO functional enrichment analysis of the intersecting genes with the top three of BP, CC and MF terms and KEGG pathways. The horizontal coordinate shows -log(p.adjust) values and the vertical coordinate shows GO terms. **(C)** The enrichment results are displayed on the network, and the node size represents the number of genes enriched. The red dots represent the nine genes that were enriched.

To explore the involvement of ER stress in sepsis, we performed a Venn analysis of the sepsis-related DEGs and ER stress-related genes (**Figure 4A**), and functionally annotated the intersecting genes by GO and KEGG analyses. As shown in **Figure 4B** and **Table 2** the genes are enriched in biological processes such as response to ER stress, negative regulation of response to ER stress, negative regulation of protein exit from the ER, cell components including platelet alpha granule lumen, platelet alpha granule, phagophore assembly site membrane, and molecular functions such as ubiquitin-like protein ligase binding, ubiquitin-protein ligase binding and protein phosphatase 2A binding. The critical functions of the DEGs include response to ER stress, ubiquitin protein ligase binding, protein processing in ER, negative regulation of protein exit from the ER and so on (**Figure 4C** and **Table 3**).

**Table 2:** GO enrichment analysis of differentially expressed genes.

Term	ID	Description	p.adjust
BP	GO:0034976	response to endoplasmic reticulum stress	4.91E-14
BP	GO:1903573	negative regulation of response to endoplasmic reticulum stress	2.07E-06
BP	GO:0070862	negative regulation of protein exit from the endoplasmic reticulum	3.83E-06
BP	GO:0035966	response to topologically incorrect protein	4.35E-06
BP	GO:1904293	negative regulation of ERAD pathway	4.35E-06
CC	GO:0031093	platelet alpha granule lumen	0.0215363
CC	GO:0031091	platelet alpha granule	0.0215363
CC	GO:0034045	phagophore assembly site membrane	0.053925509
CC	GO:0097440	apical dendrite	0.053925509
CC	GO:0005788	endoplasmic reticulum lumen	0.053925509
MF	GO:0031625	ubiquitin-protein ligase binding	8.55E-06
MF	GO:0044389	ubiquitin-like protein ligase binding	8.55E-06
MF	GO:0051721	protein phosphatase 2A binding	0.003507229
MF	GO:0043621	protein self-association	0.008115479
MF	GO:0051087	chaperone binding	0.021453686

**Table 3:** KEGG enrichment analysis of differentially expressed genes.

Term	ID	Description	p.adjust
KEGG	hsa05219	Bladder cancer	0.000968991
KEGG	hsa04141	Protein processing in the endoplasmic reticulum	0.001091676
KEGG	hsa04115	p53 signalling pathway	0.001851097
KEGG	hsa05131	Shigellosis	0.002275623
KEGG	hsa05161	Hepatitis B	0.011814754
KEGG	hsa05144	Malaria	0.021266314
KEGG	hsa01524	Platinum drug resistance	0.037234276
KEGG	hsa05210	Colorectal cancer	0.037234276
KEGG	hsa05206	MicroRNAs in cancer	0.037234276
KEGG	hsa05222	Small cell lung cancer	0.037234276

**Figure 5:**
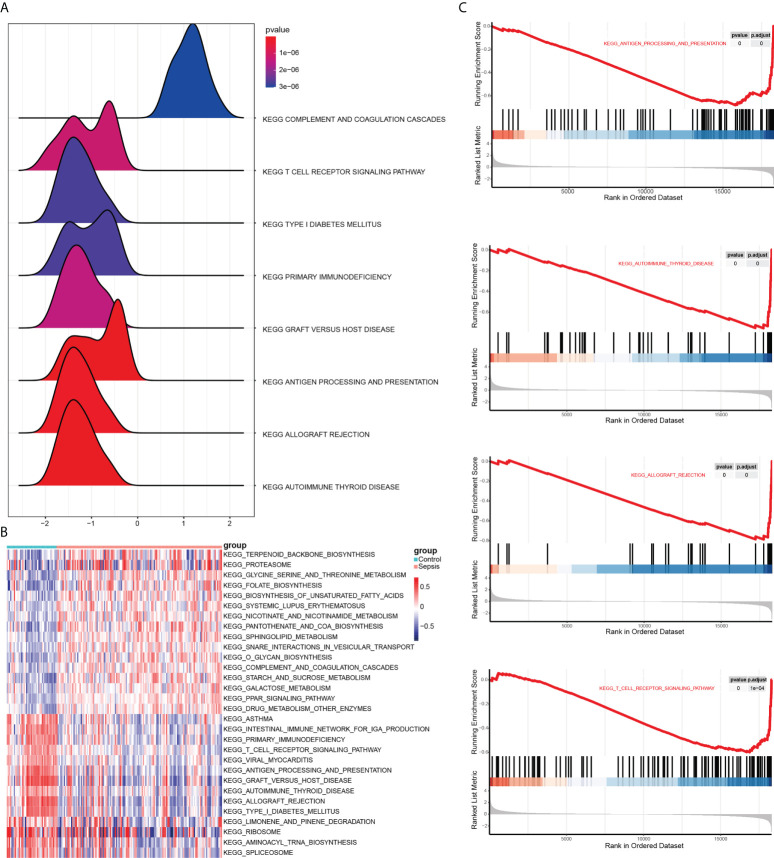
Results of GSEA and GSVA. **(A)** Mountain range plot showing the GSEA results of the merged dataset. Horizontal coordinate shows the gene ratio, vertical coordinate show the KEGG pathways, and the color indicates P-value. **(B)** Heat map showing the results of GSVA on GSEA enrichment data. Red and blue indicate activation and suppression, respectively. **(C)** The top 5 items of the GSEA.

GSEA was next performed to determine the effect of gene expression level on sepsis. As shown in **Figure 5A**, the DEGs are related to biological functions such as autoimmune thyroid disease, allograft rejection, antigen processing and presentation. The top 5 functions are shown in **Figure 5C**. To test out the enrichment results of the gene set, we used GSVA (Gene Set Variation Analysis) analysis. The expression matrix of genes among different products is transformed into the expression matrix of gene sets among samples to evaluate whether different metabolic pathways are enriched. Finally, the results are visually displayed using the pheatmap package (**Figure 5B** and **Table 4**). We found that sample grouping can distinguish the effects of gene set enrichment analysis. These results indicate activation of endoplasmic reticulum stress-related pathways is an important biological process affecting immune cell function in sepsis.

**Table 4:** GSEA analysis of differentially expressed genes GSE108474.

Description	enrichmentScore	p.adjust
KEGG_ALLOGRAFT_REJECTION	-0.787525613	1.37E-05
KEGG_GRAFT_VERSUS_HOST_DISEASE	-0.766795418	5.32E-05
KEGG_PRIMARY_IMMUNODEFICIENCY	-0.756453466	6.93E-05
KEGG_AUTOIMMUNE_THYROID_DISEASE	-0.753983298	1.72E-06
KEGG_TYPE_I_DIABETES_MELLITUS	-0.731429168	6.93E-05
KEGG_INTESTINAL_IMMUNE_NETWORK_FOR_IGA_PRODUCTION	-0.709444115	0.00011187
KEGG_ASTHMA	-0.707255846	0.007752697
KEGG_ANTIGEN_PROCESSING_AND_PRESENTATION	-0.68182909	1.72E-06
KEGG_GLYCOSPHINGOLIPID_BIOSYNTHESIS_LACTO_AND_NEOLACTO_SERIES	0.673225498	0.033512196
KEGG_STARCH_AND_SUCROSE_METABOLISM	0.671740494	0.004234432

### Identification of key ER stress-related genes in sepsis

**Figure 6:**
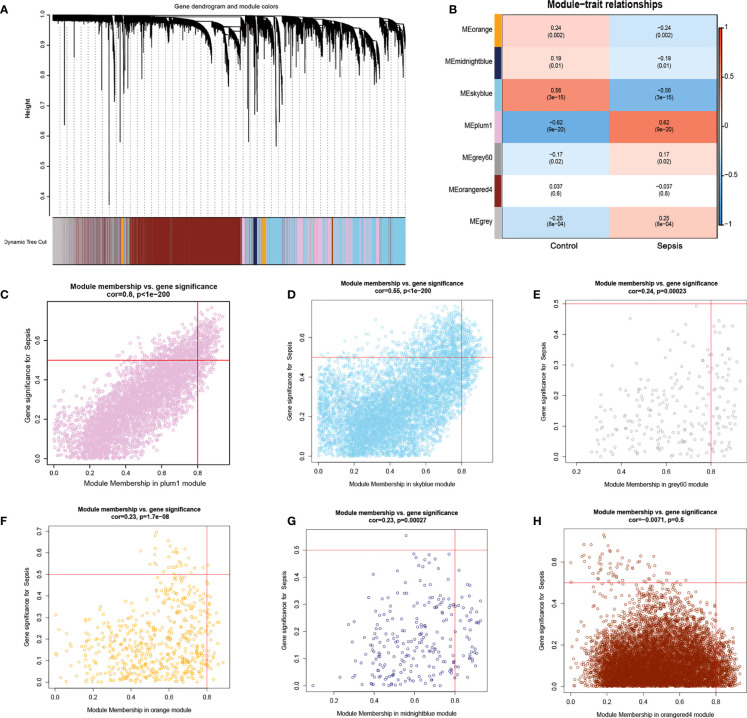
Results of WGCNA. **(A)** Cluster analysis of the combined dataset. The different module clusters are color-coded. **(B)** Correlation between the different modules in the normal and sepsis groups. **(C–H)**, Scatter diagrams for module membership vs. gene significance of sepsis. **(C)** The plum1 modules with the highest correlation. **(D)** The correlation between the skyblue module and the genes.**(E)** Display of the correlation between the grey60 module and the genes. **(F)** Display of the correlation between the orange module and the genes. **(G)** Display of the correlation between the midnightblue module and the genes. **(H)** Display of the correlation between the orangered4 module and the genes.

**Figure 7:**
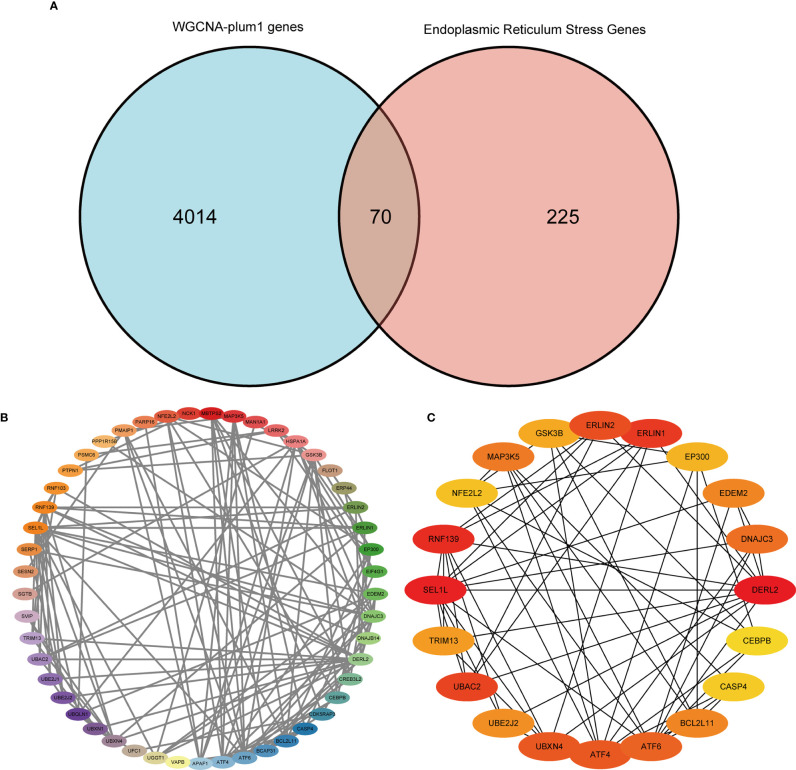
Protein-protein interaction (PPI) network. **(A)** Venn diagram showing the intersection of the most significantly correlated genes obtained by WGCNA with ER stress-related genes. **(B)** PPI network of the 70 intersecting genes. **(C)** Top 20 hub genes in the PPI network.

Furthermore, we used the WGCNA algorithm to construct co-expression modules and identify mRNA-related modules. The key parameter of soft threshold power was set to 7 to ensure the overall connectivity of the co-expression module. Seven co-expression modules were obtained and the color-coded gene clusters are shown in **Figure 6A**. The purple, gray 60 and gray modules were positively correlated with mRNA (Meplum: r = 0.62, P = 9e^−20^; Megrey60: r = 0.17, P = 0.02; Megrey: r = 0.25, P = 8e^−04^), and the orange, dark blue, sky blue and orange-red modules showed negative correlation with mRNA (Meorange: r = -0.24, P = 0.002; Memidnightblue: r = -0.19, P = 0.01; Meskyblue: r = -0.56, P = 3e-15; Meorangered: r = -0.037, P = 0.6) (**Figure 6B**). Next, the correlation of the module membership with the sepsis samples was shown (**Figures 6C–H**). The purple module was most significantly correlated to sepsis (**Figure 6C**), and its characteristic genes with the highest correlation were intersected with ER stress-related genes. There were 70 intersecting genes in the venn diagram (**Figure 7A**). PPI network analysis was performed on these genes, and those with interaction scores greater than 0.4 are shown in **Figure 7B**. The top 20 hub genes were identified with the MCC algorithm, and are shown in (**Figure 7C**). In conclusion, a multi-factor network indicated complex interaction of the 20 ER stress-related hub genes in sepsis.

### Identification of sepsis subtypes and diagnostic markers

**Figure 8:**
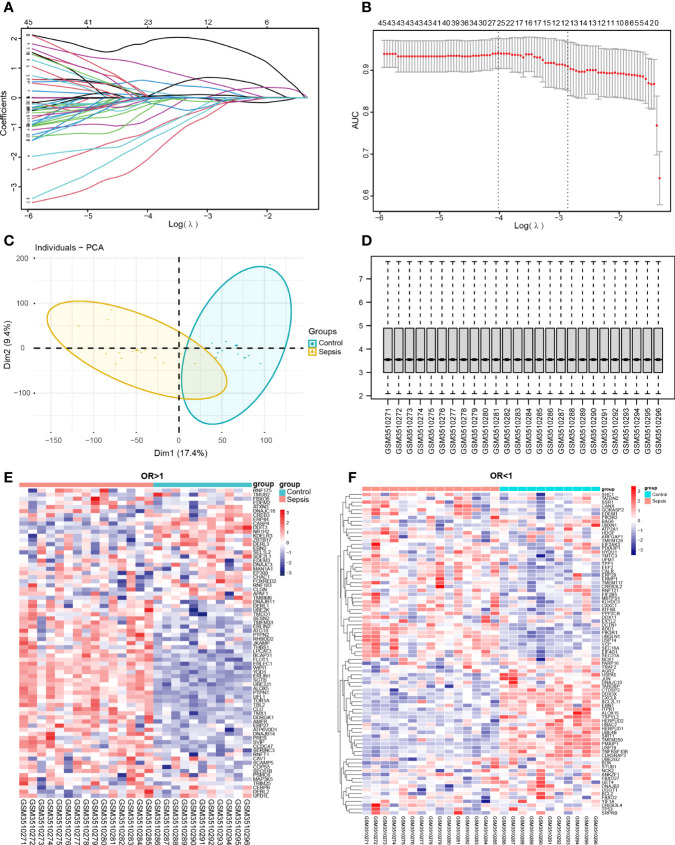
Screening for diagnostic markers. **(A, B)** Lasso analysis of the combined dataset. **(C, D)** PCA plot and box plot of the validation set GSE123729 data after correction. **(E, F)** Heat map showing differential expression of diagnostic markers in the validation set obtained by one-way logistic regression analysis. Red indicates up-regulation, blue indicates down-regulation, and darker colors indicate a larger fold change.

The potential diagnostic markers of sepsis were screened from the DEGs of the combined dataset using LASSO regression and logistic regression. As shown in (**Figures 8A, B**), there were 76 genes with OR > 1 and 85 genes with OR < 1 (and P < 0.05). The potential diagnostic markers were validated on the GSE123729 dataset by PCA, which showed that most markers distinguished sepsis from normal samples (**Figures 8C, D**). The differential expressions of these markers in the validation dataset are shown in the heat maps in (**Figures 8E, F**) and **Table 5**.

**Table 5:** Univariate logistic regression.

Character	OR	CI	P. Value
SCAMP5	7.64	1.66-35.13	0.01
DNAJC18	3.74	1.07-13.06	0.04
TARDBP	0.05	0.01-0.2	0
SDF2L1	1.98	1.11-3.55	0.02
FBXO2	0.3	0.1-0.95	0.04
FBXO6	3.09	1.84-5.21	0
TBL2	3.58	1.37-9.36	0.01
RNF175	2.77	1.3-5.87	0.01
PDIA3	0.2	0.09-0.45	0
HDGF	0.57	0.35-0.93	0.02

**Figure 9:**
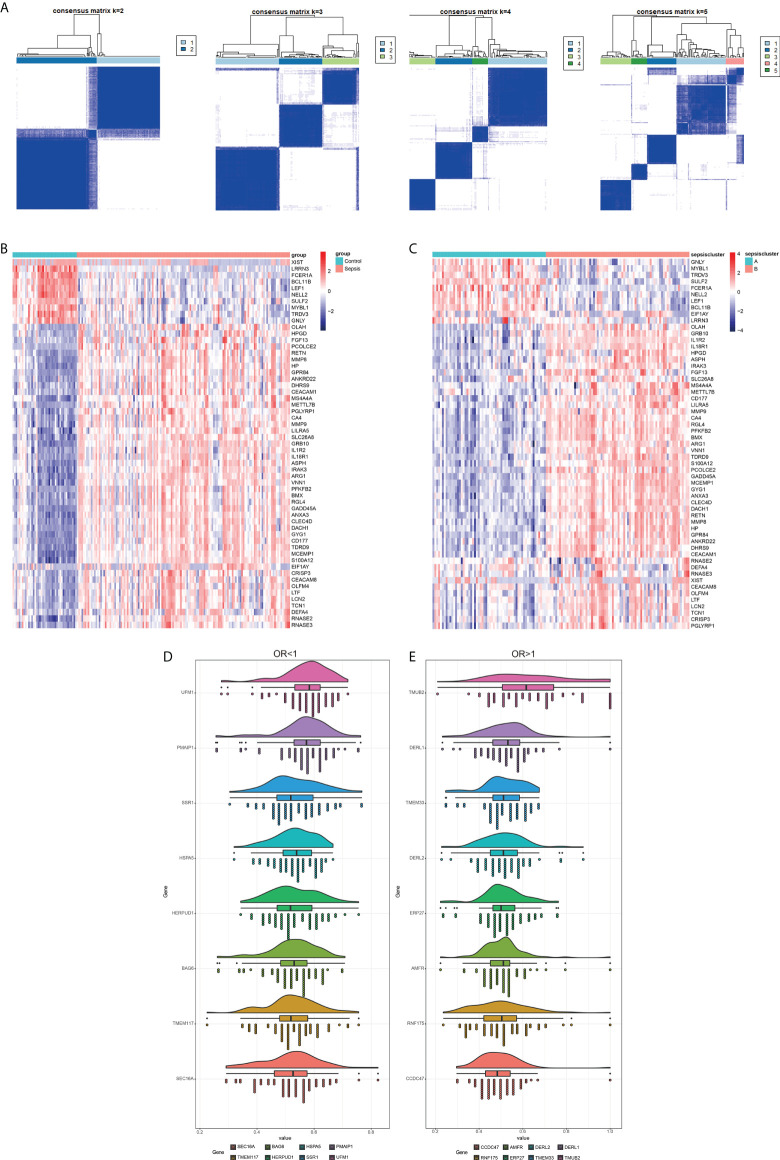
Identification of sepsis subtypes and diagnostic markers. **(A)** The number of genotype clusters in the sepsis dataset. **(B)** Heat map of diagnostic genes based on control and sepsis groups. **(C)** Heat map of diagnostic genes based on sepsis subtype. Red indicates activation and blue indicates inhibition. **(D)** Diagnostic markers with OR less than 1. **(E)** Diagnostic markers with OR more significant than 1.

Fifty-seven DEGs were significantly related to sepsis, including 47 up-regulated and 10 down-regulated genes, and were used to cluster the sepsis datasets. When the number of genotypes was set to 2, the sepsis-related genes were able to classify the sepsis samples and distinguish them from the control samples (**Figure 9A**). The heat map of these genes in the normal and sepsis groups indicated differential expression (**Figure 9B**). The sepsis subtypes were then used to map the same genes again, and the difference was more pronounced (**Figure 9C**). The diagnostic markers with OR < 1 and OR > 1 were screened to improve accuracy, and the top 8 genes with the highest correlation are shown in (**Figures 9D, E**.) Together, these results indicated that the immune-related ER stress genes achieves strong stability and high accuracy in predicting sepsis patients.

### Predictive value of SCAMP5

**Figure 10:**
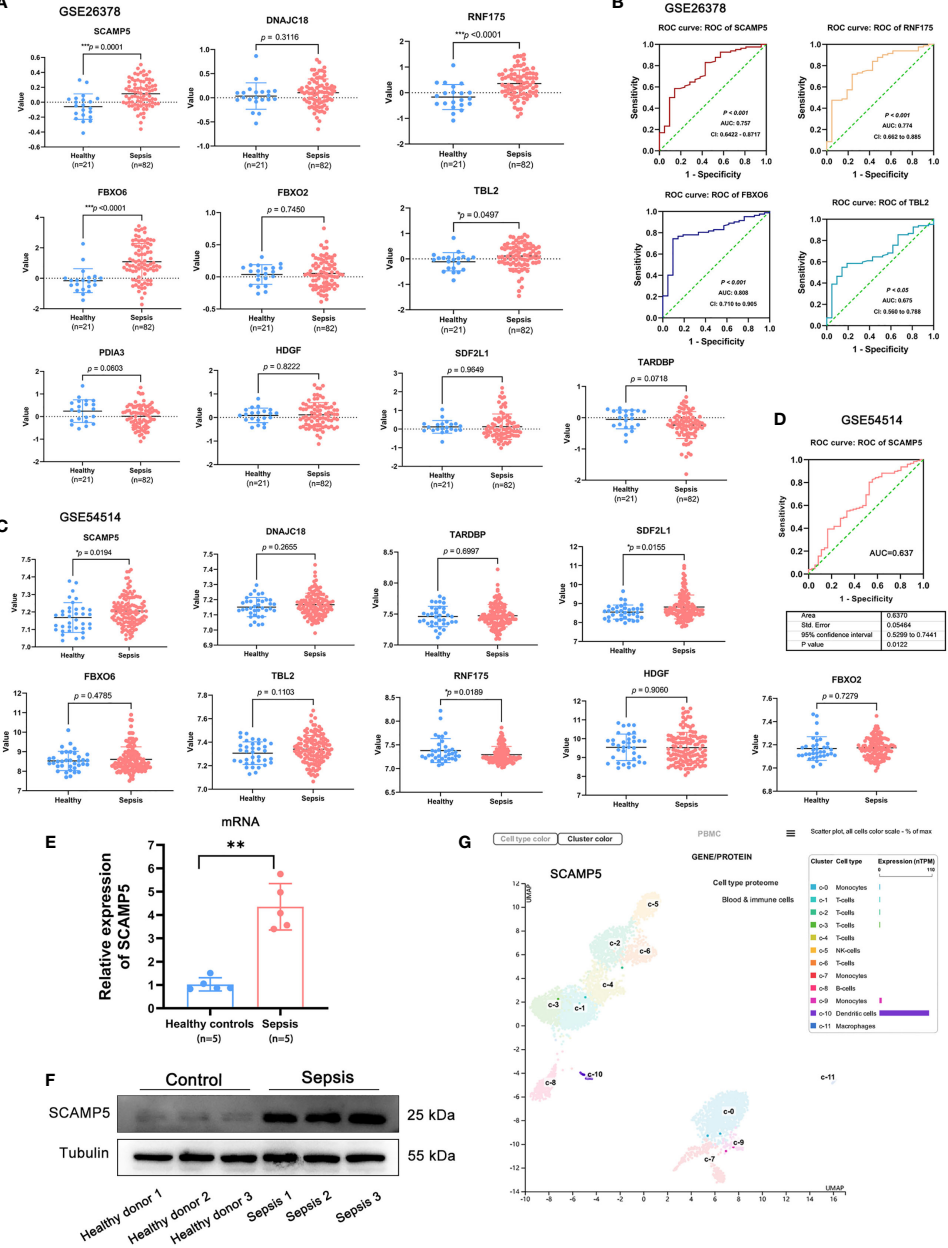
SCAMP5 is highly expressed in patients with sepsis and has significant diagnostic value. **(A)** Expression of hub genes in the control and sepsis samples in GSE26378. SCAMP5, RNF175, FBXO6 and TBL2 were significantly up-regulated in the sepsis patients (P < 0.05 by the two-sided t test. **(B)** ROC curve showing predictive value of SCAMP5 for sepsis in GSE26378 with AUC = 0.757. **(C)** Expression of hub genes in the control and sepsis samples in GSE54514. SCAMP5 and SDE2L1 were significantly up-regulated in the sepsis patients (^*^P < 0.05 by the two-sided t test). **(D)** ROC curve showing predictive value of SCAMP5 for sepsis in GSE54514 with AUC = 0.637. **(E)** SCAMP5 mRNA levels in the PBMCs from healthy controls and sepsis patients as determined by qRT-PCR. Mean ± SD (n = 5), **P < 0.01. **(F)** SCAMP5 protein levels in the PBMCs from healthy controls and sepsis patients. **(G)** Single-cell sequencing database results showing that SCAMP5 is expressed in the dendritic cells.

We further assessed the predictive value of the sepsis hub genes on the GSE26378 and GSE54514 datasets that included data of 39 healthy controls and 117 sepsis patients. SCAMP5 was significantly up-regulated in the sepsis samples compared to the control samples in both datasets (P < 0.05). On the other hand, while RNF175, FBXO6 and TBL2 showed a trend towards higher expression levels in the sepsis patients in GSE26378, no significant difference was observed in GSE54514 (**Figures 10A, C**). ROC analysis further demonstrated that SCAMP5 could accurately predict sepsis, with AUC of 0.757 in GSE26378 and 0.637 in GSE54514 (**Figures 10B, D**). We then tested the expression levels of SCAMP5 in the PBMCs from sepsis patients (n = 5) and healthy donors (n = 5), and found that SCAMP5 protein and mRNA were both up-regulated in the PBMCs from sepsis patients compared to healthy controls (**Figures 10E, F**). In addition, analysis of single-cell sequencing results in Protein Atlas database (https://www.proteinatlas.org/ENSG00000198794-SCAMP5/single+cell+type/PBMC) showed that SCAMP5 was expressed in the circulating DCs (**Figure 10G**). These results indicate that SCAMP5 is a potential diagnostic marker for sepsis.

## Discussion

Sepsis is a syndrome associated with a high mortality rate, and is therefore a serious public health concern worldwide. During the COVID-19 pandemic, some severe and critically ill patients exhibited multiple organ dysfunction that met the diagnostic criteria of sepsis (4). In recent years, the key role of immune cell apoptosis in sepsis-related immune dysfunction has been elucidated (26). Sepsis-induced apoptosis of immune cells not only leads to the depletion of critical immune effector cells, but also exerts an immunosuppressive effect (27). Some studies have also suggested a pathological role of ER stress in inflammatory diseases, including sepsis (28, 29). In addition, the ER stress-mediated apoptosis pathway is a potential therapeutic target in sepsis (30, 31).

Machine learning algorithms are increasingly being used to create decision models that aid in disease diagnosis and treatment (32). In the current study, we screened the DEGs between sepsis patients and healthy individuals, which can not only help identify potential diagnostic/prognostic biomarkers or therapeutic targets for sepsis from highly related gene aggregation modules, but also elucidate the molecular mechanisms underlying the pathogenesis of sepsis. We identified 577 DEGs from the combined GSE9960 and GSE57065 datasets, of which 325 were up-regulated and 330 were down-regulated in the sepsis samples relative to the controls.

In addition, we also observed significant differences in the type and abundance of infiltrating immune cell populations between the two groups, which underscores the role of immune cells in the development of sepsis. Monocytes and macrophages are instrumental to the pathophysiological process of sepsis and inflammation (33). The systemic inflammatory response elicited by the circulating innate immune cells during sepsis also influences the tissue-resident immune cells, which can compromise the functions of vital organs (34). Sepsis development is also associated with significant lymphopenia, which is characterized by decreased counts of CD8^+^ and CD4^+^ T cells, B cells and natural killer (NK) cells (35). Furthermore, burn patients with sepsis have significantly higher numbers of circulating DCs compared to burn patients without sepsis (36). In our study, B cells, NK cells, T cells and DCs were much more abundant in the sepsis samples compared to the controls, and therefore may play a crucial part in establishing the immune microenvironment about sepsis.

Our studies indicate that the immune cell dysfunction in sepsis is closely related to ER stress. Functional annotation of the sepsis-related DEGs indicated significant enrichment of biological process, molecular functions, cell components, biological pathways and diseases involving ER stress. A recent study has also revealed that there is a fascinating and novel interaction between ER stress with sepsis-associated cell death (37, 38). ER stress is also a trigger for apoptosis, except for mitochondrial apoptotic pathwaysand death receptor (39, 40). ER function is disrupted during sepsis, resulting in acute or chronic ER stress, which may initiate apoptosis in the damaged cells (41). Thus, ER stress-mediated apoptosis pathway may be a novel therapeutic target against sepsis-induced immune cell apoptosis (42).

We also screened for potential diagnostic markers for sepsis among the significant DEGs, and validated them in the GSE123729 dataset. The hub genes that can distinguish sepsis from normal samples were identified, which included *SCAMP5*, *DNAJC18*, *TARDBP*, *SDF2L1*, *FBXO2*, *FBXO6*, *TBL2*, *RNF175*, *PDIA3* and *HDGF*. Secretory carrier membrane protein 5 (SCAMP5) is an integral membrane protein that was highly expressed in the sepsis samples compared to the controls. SCAMP5 is known to be brain specific which is involved in vesicle transport (43). Recent studies show that SCAMP5 is a candidate biomarker gene for autism and its downregulation is related to the synaptic dysfunction in autistic patients (44). Moreover, F-box protein 6 (FBXO6) is a subunit of the ubiquitin protein ligase complex, which bind to glycosylated substrates within F-box-associated domains in endoplasmic reticulum (ER) stress-associated degradation (45). Phosphorylation of TBL2 by ATM/ATM in response to DNA damage identifies TBL2 is considered to be a member of the cellular oxidative damage response network, as it phosphorylated by ATM/ATM in response to DNA damage (46). We confirmed the high expression levels of SCAMP5 mRNA and protein in PBMCs isolated from sepsis patients. Moreover, SCAMP5 was expressed in the peripheral DCs as per the single-cell sequencing results from the Protein Atlas database. Taken together, these findings suggest that SCAMP5 is a potential diagnostic marker for sepsis, and may play a vital role in its development. However, it is worth noting that the diagnosis and prediction of SCAMP5 sepsis still need further validation in clinical trials with large sample size. Meanwhile, the regulatory role of SCAMP5 in immune-related ER stress needs to be further investigated in functional and mechanistic studies.

To summarize, we developed a stable and accurate signal to evaluate the diagnosis of sepsis through integrated bioinformatics and machine learning algorithms. This prediction model can surveillance protocols and optimize decision-making for individual sepsis patients. Moreover, SCAMP5 was preliminarily identified as a key driver of sepsis that may affect its progression by regulating ER stress. The diagnostic and therapeutic potential of SCAMP5 in sepsis warrants further investigation.

## Data availability statement

The datasets presented in this study can be found in online repositories. The names of the repository/repositories and accession number(s) can be found in the article/supplementary material.

## Ethics statement

The collection of blood samples from human subjects was approved by the Medical Ethics Committee of Shenzhen Hospital of Southern Medical University (Shenzhen, China, approved ID: NYSZYYEC20200039). The clinical data is available at the China Clinical trial Registration Center (No. ChiCTR2100043761). The patients/participants provided their written informed consent to participate in this study.

## Author contributions

YTL and CC performed study concept and design; TG, YBL, and SY performed research and writing the paper; TG, YBL, HG, and ZT performed the experiments; TG, ZT, HG, MZ, and ZP provided acquisition, analysis and interpretation of data, and statistical analysis; YTL and TG supervised research, review and revision of the paper. All authors read and approved the final manuscript.

## Funding

This study was supported by the National Natural Science Foundation of China (82002087, 82072215), Science and Technology Planning Project of Shenzhen, China (JCYJ20210324134602008, JCYJ20210324134602006) and the Research Foundation of Shenzhen Hospital of Southern Medical University (PY2020YM01).

## Conflict of interest

The authors declare that the research was conducted in the absence of any commercial or financial relationships that could be construed as a potential conflict of interest.

## Publisher’s note

All claims expressed in this article are solely those of the authors and do not necessarily represent those of their affiliated organizations, or those of the publisher, the editors and the reviewers. Any product that may be evaluated in this article, or claim that may be made by its manufacturer, is not guaranteed or endorsed by the publisher.
